# Computational Analysis of the Optical and Charge Transport Properties of Ultrasonic Spray Pyrolysis-Grown Zinc Oxide/Graphene Hybrid Structures

**DOI:** 10.1186/s11671-016-1466-x

**Published:** 2016-05-12

**Authors:** Amgad Ahmed Ali, Abdul Manaf Hashim

**Affiliations:** Malaysia-Japan International Institute of Technology, Universiti Teknologi Malaysia, Jalan Sultan Yahya Petra, 54100 Kuala Lumpur, Malaysia

**Keywords:** Molecular orbital (MO), Graphene oxide, Spray pyrolysis, Zinc oxide, Density functional theory, Nanostructure

## Abstract

We demonstrate a systematic computational analysis of the measured optical and charge transport properties of the spray pyrolysis-grown ZnO nanostructures, i.e. nanosphere clusters (NSCs), nanorods (NRs) and nanowires (NWs) for the first time. The calculated absorbance spectra based on the time-dependent density functional theory (TD-DFT) shows very close similarity with the measured behaviours under UV light. The atomic models and energy level diagrams for the grown nanostructures were developed and discussed to explain the structural defects and band gap. The induced stresses in the lattices of ZnO NSCs that formed during the pyrolysis process seem to cause the narrowing of the gap between the energy levels. ZnO NWs and NRs show homogeneous distribution of the LUMO and HOMO orbitals all over the entire heterostructure. Such distribution contributes to the reduction of the band gap down to 2.8 eV, which has been confirmed to be in a good agreement with the experimental results. ZnO NWs and NRs exhibited better emission behaviours under the UV excitation as compared to ZnO NSCs and thin film as their visible range emissions are strongly quenched. Based on the electrochemical impedance measurement, the electrical models and electrostatic potential maps were developed to calculate the electron lifetime and to explain the mobility or diffusion behaviours in the grown nanostructure, respectively.

## Background

Two-dimensional (2D) sheet of sp^2^-hybridized carbons known as graphene has attracted great attention because of its exceptional optical, electrical, chemical and mechanical properties that impose promising ability for developing new generation of functional nanomaterials for various applications [1–3]. An ideal graphene nanosheet is found to possesses more light transmittance, flexibility and conductivity than indium tin oxide or single-wall carbon nanotubes for flexible transparent conductor or electrode applications [2, 3]. Lately, various methods are reported for growing graphene in large area size. In particular, chemical vapour deposition (CVD) is considered as the most common method for preparing high-quality large-area graphene due to the excellent controllability of thicknesses of the grown layers.

Concerning the targeted applications, there have been extensive efforts to combine the unique properties of graphene with metal-oxide nanostructures, namely, zinc oxide (ZnO) nanostructures to realize a novel hybrid structure for new generation of electronic, optoelectronic and photovoltaic applications [1, 4–8]. For instance, nanorods (NRs) and nanowires (NWs) as prolonged nanostructures have privileges over other structures. The optical reflectance properties of NRs are much better than thin films, thus significantly its absorption increases, which is particularly interesting for photovoltaic and photon-induced hydrophillicity applications.

Recently, intensive works have been conducted in developing ZnO/graphene hybrid structures either by vapour-phase [9–12] or liquid-phase techniques [13–17]. Recently, we report the evolution of ZnO nanostructures grown on graphene using a low-temperature ultrasonic-assisted spray pyrolysis technique [18, 19]. The effects of pyrolysis parameters, i.e. growth temperature, precursor injection/growth time, precursor molarity and precursor flow rate, on the grown structures were investigated. The growth modelling and process optimization was carried out to explain the observed evolution of ZnO nanostructures. The responses, i.e. structure density, structure shape factor and structure size, were evaluated. The modelling and optimization of the ultrasonic spray pyrolysis parameters for the growth of ZnO nanostructures on graphene layer using the response surface methodology (RSM) method were discussed. In this article, we report the computational analysis of the measured optical and charge transport properties of the spray pyrolysis-grown ZnO nanostructures. Most of the literatures regarding ZnO/graphene hybrid structures have mainly focused on the discussions of the experimentally measured morphological, structural and optical properties without any well-established computational analysis to explain the measured optical and charged particle transport characteristics [5, 7–9, 14, 20].

## Methods

It is worth to summarize the experimental conditions and method for the growth of ZnO nanostructures on graphene. Single-layer graphene on a SiO_2_ (285 nm)/Si wafer (Graphene Laboratories, USA) was used as a substrate. The size of samples used for the growth is 1.0 × 1.0 cm^2^. Zinc acetylacetonate hydrate powder, Zn(C_5_H_7_O_2_)_2_ xH_2_O (Sigma-Aldrich), was used without any further purification. Other chemicals, such as solvents and reagents, were research grade and used as received. The substrates were cleaned with ethanol and vacuum dried at 60 °C prior to the growth. First, a substrate was heated to the required temperature under 35-mbar vacuum. Then, ZnO liquid precursor (zinc acetylacetonate in ethanol solution) was supplied to the ultrasonic atomizer at the desired flow rate at various mixing ratios with ethanol before being sprayed onto the substrate surface. Because an ultrasonic nozzle was used to atomize the solution into nanodroplets, only low temperature was required for the vapourization of the droplets. The response surface methodology (RSM) to control the deposition parameters, i.e. precursor flow rate, molarity, substrate temperature and deposition time, utilizing the Box-Behnken model, was used with the other screening techniques as well to optimize the responses, i.e. structure density, structure shape factor and structure size. Table 1 summarizes the deposition parameters and structural responses used in the present study. It is worth noting that the chosen four experimental runs were referred to the deposition parameters developed in the previous study [18].

**Table 1:** Deposition parameters and structure properties

Deposition parameters					Properties	
Run (*R*_i_)	Flow rate (ml/min)	Temp. (°C)	Deposition time (min)	Molarity (M)	Density %	Size nm
1	8	500	0.5	0.4	52.33	25.6
2	8	210	5	0.4	77.51	28.0
3	1	355	38	0.05	84.54	30.5
4	0.05	210	30	0.4	99.17	10.6

All experimental runs were carried out under a fixed pressure of 35 × 10^−4^ mbar. Nitrogen gas was used for scavenging any volatile contaminant before spraying took place as well as to act as a carrier gas. Morphological and element compositional characterization was carried out using field-emission scanning electron microscopy (FESEM) equipped with energy-dispersive X-ray spectroscopy (EDS) facilities.

The optical absorption properties of the grown nanostructures were measured using UV-vis spectroscopy (Perkin Elmer/Lambda 35). Furthermore, the photoluminescence properties of the ZnO/graphene nanostructures excited at a wavelength of 335 nm at room temperature were recorded using fluorescence analyser (WITec Alpha300 M). The setup of this instrument combines the advantages of confocal and near-field optical microscopy together. Moreover, in order to investigate the possible structural defects that affect the absorption properties of the obtained structures under UV light excitation, a time-dependent density functional theory simulation (TD-DFT) was performed. The ωB97X-D functional was used to calculate the UV-vis spectrum for a variety of possible structural defects in ZnO lattice grown on single layer graphene. The Spartan 14 quantum chemistry package (Wavefunction, USA) was used to perform all calculations in this study [21, 22]. Equilibrium geometries were optimized by the B3LYP density functional method using the 6-311G** basis set; the developer of Spartan chose the Gaussian exponents for polarization functions to give the lowest energies for the modelled molecules. The polarization of the *s* orbitals on hydrogen atoms is crucial to accurately describe the bonding in acetylacetonate systems, particularly the hydrogen bonding. Furthermore, the 6-31G** basis set provides the *p*-type polarization functions for hydrogen. This can improve the total energy of the system along with the results for systems with large anions and can impose more flexibility [21, 22]. Zn-containing structures were also optimized with larger basis sets and higher levels of theory [21], where all correction energies were calculated using the 6-311G**, 6-311++G** and 6-311++G(2df,2pd) basis sets. The calculations involving anions and absolute acidity need to be carefully treated especially in selecting the basis sets since the excess electrons are weakly coupled to specific atoms or groups of atoms. The basis sets should provide diffuse *s*- and *p*-type functions on non-hydrogen atoms. This is usually designated by the ‘+’ sign, as in 6-311++G**. The second ‘+’ sign indicates that a diffuse function is added to hydrogen [21, 22].

To obtain more accurate energy calculations, single-point calculations were performed at the B3LYP/6-311G** optimized geometry using the B3LYP/6-311+G**, MP2/6-311+G**, B3LYP/6-311+G(2df,2p) and MP2/6-311+G(2df,2p) levels of theory at ground state before studying the excited states. Since the present study focuses on the optoelectronic properties of the ZnO/graphene structures, a graphene unit cell was chosen to represent the graphene at the interface layers boundary. Such assumption is made based on the fact that the optical and electronic properties calculated at the interface boundaries are lattice independent. The DFT calculations for such case are only considering the electronic structure of single atom regardless the atom arrangement in lattice. For instance, the electrostatic potential *ε*_p_, is calculated as the energy of interaction of a positive point charge located at p with the nuclei and electrons of a molecules. *ε*_p_ is calculated using the following Eq. 1 [21].

1 $$ {\varepsilon}_p={\displaystyle \sum_A^{\mathrm{nuclei}}\frac{{\displaystyle {Z}_A}}{{\displaystyle {R}_{\mathrm{AP}}}}-{\displaystyle \sum_{\mu}^{\mathrm{basis}}{\displaystyle \sum_{\nu}^{\mathrm{functions}}{P}_{\mu v}{\displaystyle \int \frac{\varphi_{\mu }(r){\varphi}_{\nu }(r)}{r_p}dr}}}} $$

Here, the first summation is over nuclei A. *Z* is the atomic number, and *R*_AP_ is the distance between the nuclei and the point charge. The second pair of summation is over basis functions, *φ*_i_. *P* is the density matrix, and the integral reflects the Columbic interactions between the electrons and the point charge. *r*_p_ is the distance separating the electron and the point charge. A surface for which the electrostatic potential is negative (a negative potential surface) delineates regions in a molecule which are subject to electrophilic attack. The bonds will be made to centres for which the spin density is the greatest [21].

The electrochemical impedance spectroscopy (EIS) measurements were performed to study the transport of charged particles through the synthesized ZnO/graphene hybrid structures. A platinum electrode was used as a current electrode (CE), and glass electrode filled with 3 mol/l KOH reference electrolyte solution was used as a reference electrode (RE). The specimen (0.5 × 1.0 cm^2^) was fitted onto copper sheet and connected to the working electrode (WE) clamp. The three electrodes were then plugged to Autolab potentiostat (PGSTAT128N/FRA32M, Metrohm). Zinc nitrate solution was used as an electrolyte with 0.4 M concentration. A low-amplitude sinusoidal excitation signal (voltage range of −0.015 to 0.015 V) was then introduced to the cell at frequency range from 0.1 to 10^5^ Hz.

## Results and Discussion

### Morphological Properties

**Fig. 1:**
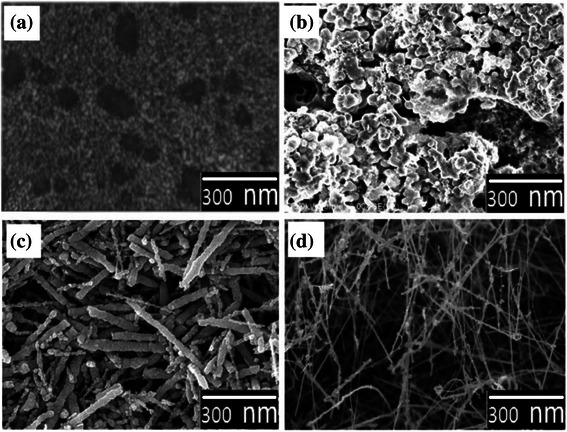
The *top view* FESEM images of structures obtained from the experimental run. **a** R1 (polycrystalline ZnO non-continuous thin film structure), **b** R2 (ZnO NSCs), **c** R3 (ZnO NRs) and **d** R4 (ZnO NWs)

Figure 1a–d represents the FESEM images that resulted from the experimental runs R1 to R4, respectively. Figure 1a demonstrates polycrystalline ZnO non-continuous thin film-like structure where the grains tend to aggregate in irregular-shaped islands with relatively large boundaries. Figure 1b shows a high-density phase of ZnO nanosphere clusters (NSCs). Furthermore, Fig. 1c shows a high-density ZnO NR-like structure with relatively small diameters. Finally, Fig. 1d shows a high-density ZnO NW-like structure with obviously fine diameters. Here, the nanostructure densities were determined as an average weight percent of ZnO nanostructures [23, 24] through five EDS scans performed at five different locations in the samples. Whereas, the nanostructure size was determined by calculating the average diameter of a circle that was a tangent to the outer profile of the nanostructures using three FESEM images taken at five different locations in the sample. The details of the growth mechanism and structural properties of the grown structures can be also obtained in [18].

As presented in [18], by changing the flow rate, growth time, substrate temperature and molarity, diverse groups of nanostructures in terms of shape, size and density were able to be grown. As discussed in the same report, the evolution of ZnO structures was well explained by our developed modelling approach which enables a precise prediction on the structure to be grown.

### Optical Properties

**Fig. 2:**
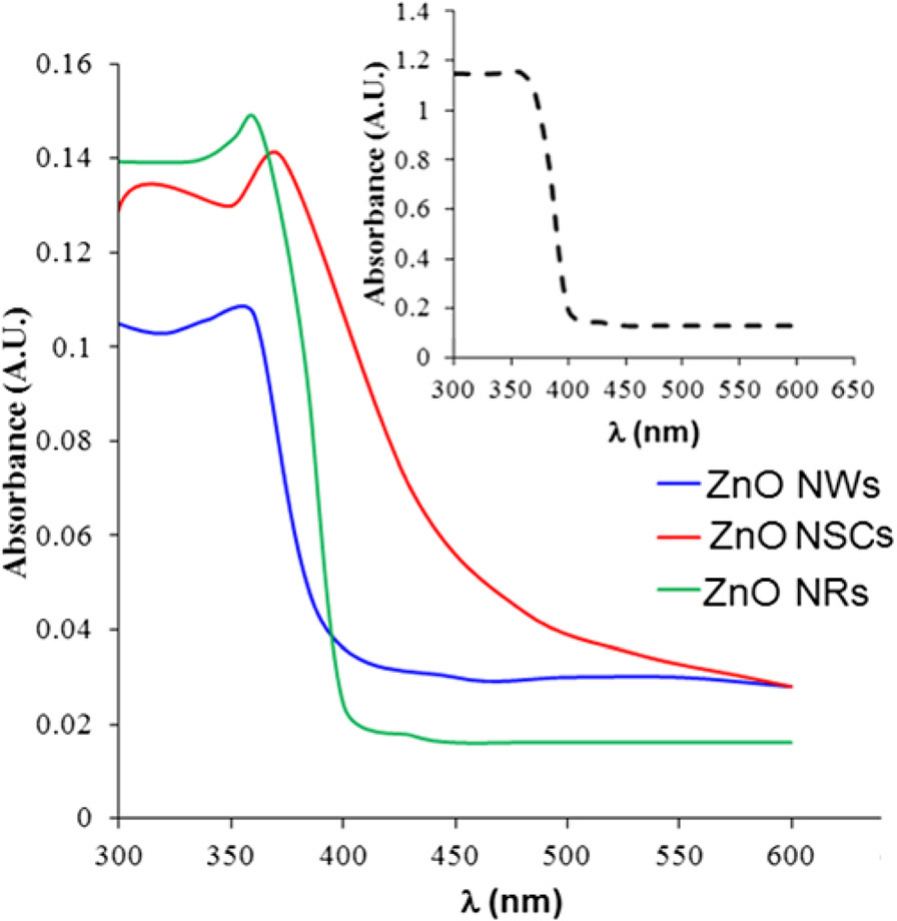
The optical absorption spectra of ZnO nanostructures on graphene samples. *Inset* is a spectrum of a reference ZnO thin film

Figure 2 shows the optical absorption spectra of the grown ZnO NSCs, NRs and NWs obtained by the experimental R2, R3 and R4, respectively, since we are interested on the properties of the nanostructures. Here, a standard ZnO polycrystalline film was used as a reference. The reference ZnO film shows a broad and strong adsorption with maximum at 378 nm. On the other hand, the absorption peak shifted to 363 nm for the ZnO NRs and 368 nm for the ZnO NWs. It can be said that such 8–15 nm blue shift is a common property for the 1D ZnO structures. This blue shift observed may be attributed to the quantum confinement effect resulted from the size-reduced crystal of the nanoscale ZnO structures [19, 25]. These previous studies investigated the relationship between the UV emission of ZnO nanostructures and the effect of stress on the energy bandgap [19, 25].

The change of the energy bandgap in the as-grown ZnO nanostructures can be used to prove the rearrangement of the band structure and to provide information on the stress [19, 25]. Here, the upward shift in the energy bandgap corresponds to the occurrence of compressive stress in the crystal, whereas the narrowing of energy levels is found to be resulted from the residual tensile stress. The shift in the optical phonon mode of the ZnO nanoparticles indicates the effect of stress on the wurtzite structure. All these studies indicate that oxygen content in addition to the residual stresses can affect the optical properties of ZnO nanostructures [19, 25].

**Fig. 3:**
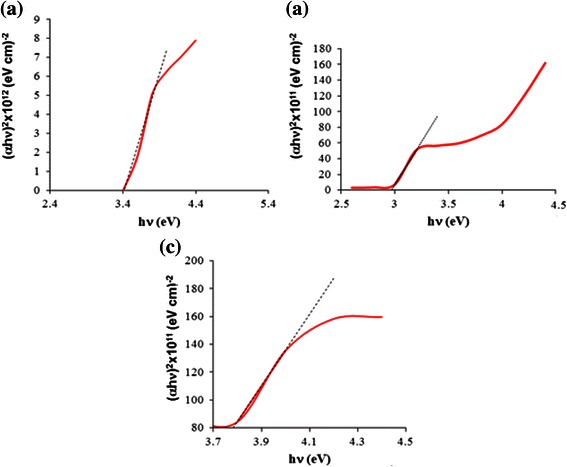
The (*αhν*)^2^ vs. (*hν*) for **a** NW-, **b** NR- and **c** NSC-based heterostructures

On the other hand, the ZnO NSCs show a red shift where its excitonic peak shifted to 388 nm. However, it is still in the UV range. It can be observed that its absorption edge is gradually extended to the visible light region, suggesting that it could be generally attributed to the elevated induced stresses between the NSCs. These residual stresses estimated using the lattice constants obtained from XRD data and calculated based on the strain model for hexagonal crystals may result in the changes of energy band structure, which subsequently lead to the narrowing of the gaps between the energy levels [19, 25]. The band gaps of the samples were estimated and shown in the Tauc plots (Fig. 3). The absorption coefficient, *α*, for direct inter-band transitions is given by Eq. 2.

2 $$ \alpha =A\frac{\left(h\nu -{\left.{E}_g\right)}^n\right.}{h\nu } $$

Here, *α* is the absorption coefficient, *E*_g_ is the absorption band gap and *A* is the constant depending on the transition probability. *n* value depends on the nature of the transition, i.e. allowed direct, allowed indirect, forbidden direct and forbidden indirect. For ZnO with direct band gap, the value of *n* is 2 [7]. *A* was calculated using Eq. 3.

3 $$ A=\left(\frac{2.303\times \mathrm{Abs}}{t}\right) $$

Here, Abs is the absorbance and *t* is the ZnO array thickness. The Tauc plot was generated by plotting *(αhν)*^*2*^ vs. *(hν)* as shown in Fig. 3. The band gap was estimated by the extrapolation of the linear part of the curve at (*α*h*ν*)^2^ equals to zero. It can be seen in Fig. 3a for the NW-based heterostructure that the direct band gap was found to be 3.40 eV, while for the NR-based heterostructure, the band gap was reduced to 2.90 eV as shown in Fig. 3b. Furthermore, the band gap for the NSC-based structure was found to reduce to 3.78 eV as shown in Fig. 3c, which could be attributed to the narrowing of the gaps between the energy levels caused by the red shift effect in correlation with the existence of lattice defects, i.e., oxygen vacancies. The estimated band gaps were confirmed to be in good agreement with the published results [8, 19].

**Fig. 4:**
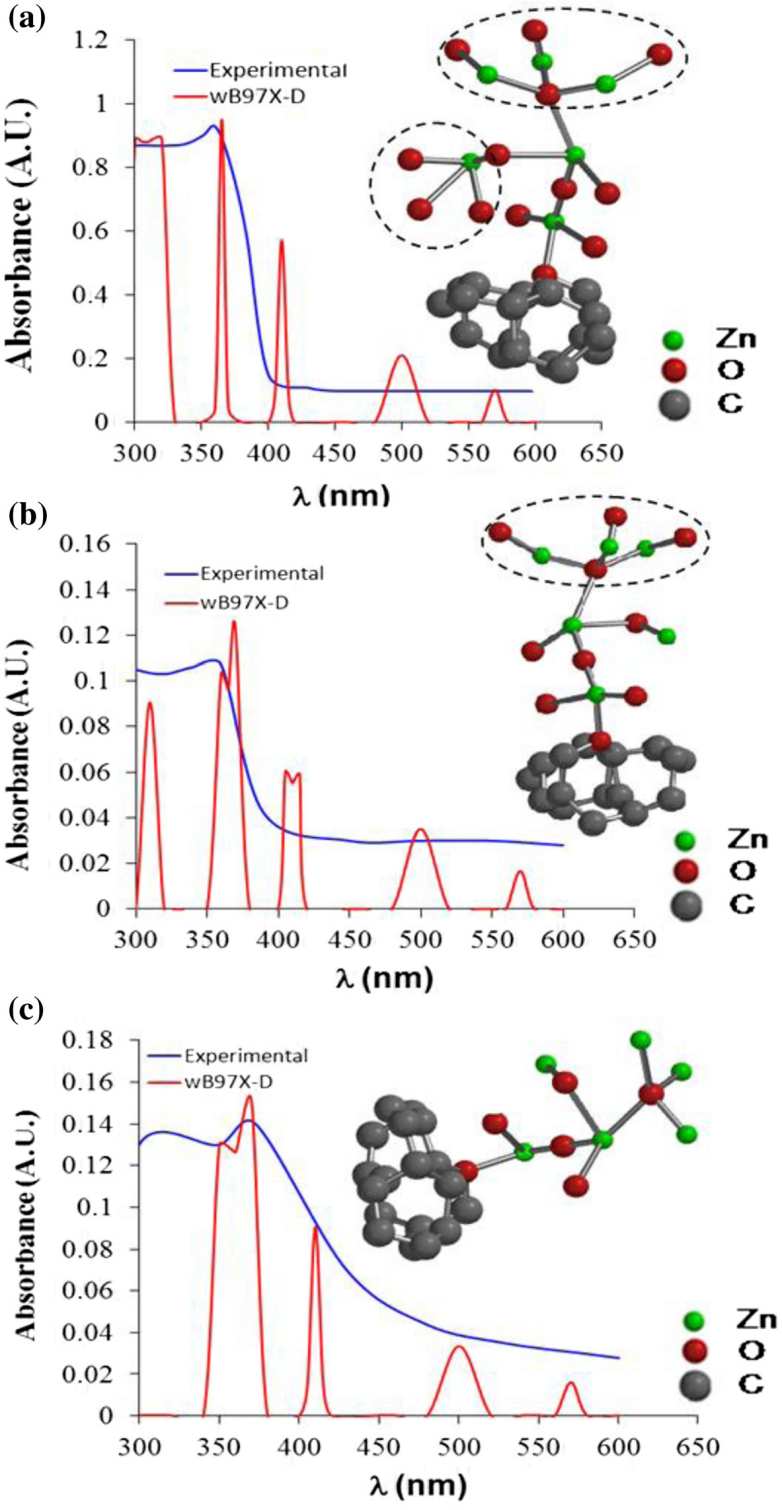
Comparison between the calculated and measured UV-vis spectra and their corresponding atomic models for **a** NW-, **b** NR- and **c** NSC-based heterostructures

In order to investigate the possible structural defects that is assumed to affect the absorption behaviour of the samples under UV light, a TD-DFT is performed. This simulation uses ωB97X-D functional to calculate the UV-vis spectrum for various possible structural defects in ZnO lattice grown on single-layer graphene. The calculated spectra are then compared to the measured ones, and its corresponding atomic model is used to speculate the structural defects. Figure 4 presents the comparison between the calculated and measured UV-vis spectra and the corresponding atomic model for the respective structures. It is worth noting that the simulation results show acceptable agreement with the measured spectra where their average similarities are above 95.2 %.

The reason of mismatch is attributed to the ability of the DFT calculations to take into account the spinning condition of electrons at excited states for complex oxides. This is mainly because of the redundancy of iteration in the density matrix caused by the spinning direction vector. Here, the percentage of similarity is calculated as the ratio of the area under both calculated curve (red) and measured curve (blue). On the other hand, the average peak position matching ratio between TD-DFT calculations and experiments is found to be around 97.5 %. Thus, the presented atomic models resulted from this simulation could be considered valid for explaining the structural defects that lead to the shown absorbance behaviour under UV light.

As shown in Fig. 4a, the ZnO NW-based structure contains excessive amount of O atoms (red spheres) in its lattice as compared to the perfect wurtzite structure [24]. It can be seen that the side Zn atom (green spheres) at the ZnO branch is covalently bonded to three extra O atoms with single bond (denoted by dashed circle). Besides, the top edge Zn atoms of the branch are singly bonded to three O atoms per each (denoted by dashed oval). Meanwhile, by considering the ZnO NR-based structures, it involves less content of O atoms as compared to the ZnO NW structure as shown in Fig. 4b. However, by comparing it to the perfect ZnO wurtzite, NR structure still contains three more O atoms (denoted by dashed oval) as can be seen in Fig. 4b. Finally, the ZnO NSC arrays show lack of O atoms inside the ZnO lattice as shown in Fig. 4c. It was reported in literature that O defects have great influences on the optical properties of the ZnO structures [28], and controlling these defects can lead to remarkable enhancement in the optoelectronic properties of the material especially for the photocatalytic ability [27, 28].

**Fig. 5:**
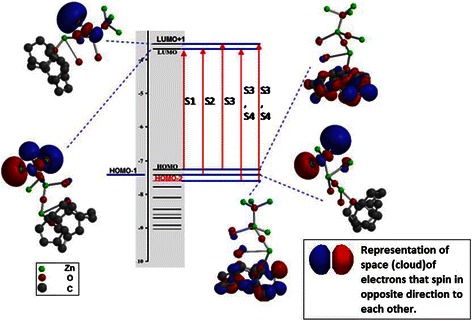
Energy levels diagram for the ZnO NSCs structure at the excited states

The common method in reducing the O defects is by annealing the grown ZnO structures in the presence of O_2_ stream [27]. However, in the present study, the treatment of the defects has been achieved by controlling the parameters of the pyrolysis process itself. For instance, the induced stresses in the ZnO NSCs lattice during the pyrolysis had caused a narrowing of the gap between the energy levels as mentioned previously. Such changes are emphasized by the molecular orbital (MO) study presented in Fig. 5, where the red and blue areas refer to the clouds of electrons spinning in the opposite direction to each other. As can be seen in Fig. 5, the gap between energy levels is reduced in the conduction band and the valence band. However, the band gap itself is found to be around 3.8 eV which is in good agreement with the measured one. Such large band gap increases the recombination rate of the charge and that is the possible reason why the adsorption edge is being dragged to the visible range [7]. This can be attributed to the minute differences in energy between the four captured excited states S1, S2, S3 and S4. In fact, the S1, S2 and S3 excited states are captured at 560, 500 and 410 nm, respectively.

It is found that S1 is the state where the charges travel from the highest occupied molecular orbital (HOMO) to the lowest unoccupied molecular orbital (LUMO) as a result of excitation. As can be seen in Fig. 5, the LUMO orbitals are localized at the graphene matrix while the HOMO orbitals are mainly localized at the free end of the ZnO branch. The single bonded O atoms at the side of the branch also have LUMO orbitals. Thus, the distance to be travelled by electrons seems to be long and that is the main cause of the large band gap.

Concerning S2, it is the state where the electrons need to travel from HOMO-1 to LUMO after being subjected to an excitation that exceeds 3.9 eV. It is shown in Fig. 5 that the HOMO-1 orbitals are localized on the ZnO branch rather than the graphene matrix; this makes the distance travelled by electrons becomes shorter. However being HOMO-1 electrons make it higher in energy than HOMO. Similar scenario repeats with S3 and S4; however, in this case, the HOMO-2 orbitals are distributed much better all over the structure which enhances the transfer of excited atoms. Such enhancement in orbital distribution at HOMO-1 and HOMO-2 is the main cause of the narrowing of the gaps between energy levels which is caused by process-induced stresses and lead to a gradual spectrum as shown in Fig. 4c.

**Fig. 6:**
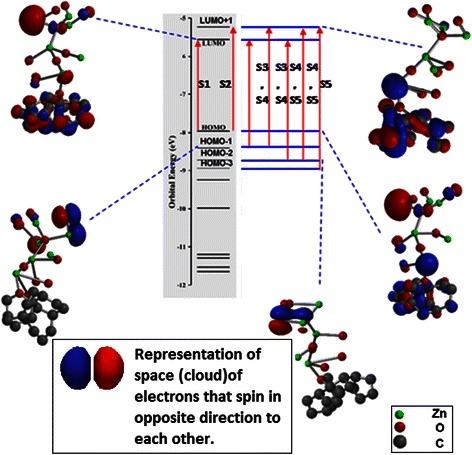
Energy levels diagram for the ZnO NRs structure at excited state

On the other hand, ZnO NRs show homogeneous distribution of the LUMO and HOMO orbitals all over the entire heterostructure as shown in Fig. 6. Such distribution contributes to the reduction of the band gap to 2.8 eV, which is in a good agreement with the experimental results. As shown in Fig. 6, LUMO orbitals are distributed all over the ZnO branches due to the existence of excessive O atoms. Here, the double-bonded O atoms to the Zn atom near to the graphene matrix lead to the charge redistribution over the graphene matrix (by back π − π* donation) and hence enable the LUMO orbitals to be localized onto graphene matrix.

It can be seen in Fig. 6 that HOMO-1 and HOMO-2 are delocalized from the graphene matrix. This can be observed from the red and blue areas that exist only at graphene matrix in atomic model, corresponding to energy levels of LUMO, LUMO + 1 and HOMO, while disappear in other energy levels in the graphene matrix. Due to the proper distribution of the LUMO and LUMO + 1 all over the heterostructure provided by the graphene layer, it allows the transfer of charges during the excitation states of S3, S4 and S5 with energy barrier less than 4 eV. Thus, it can be said that the localization of LUMO at graphene matrix lead to a better charge transfer from graphene side to ZnO end which results in the reduction of the band gap and the increase of the lifetime of the transporting charge. Consequently, the charge and hole recombination rate is expected to be enhanced.

**Fig. 7:**
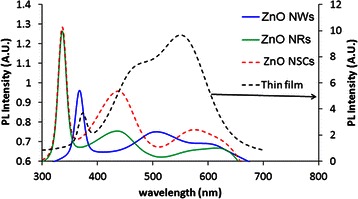
Emission spectra of ZnO/graphene hybrid structures

The photoluminescence spectra of the ZnO/graphene nanostructures, i.e. NWs, NRs and NSCs, excited at a wavelength of 335 nm at room temperature are presented in Fig. 7. In general, the peaks found in the range of 340–470 nm can be corresponded to the radiative transition of the excited electrons from occupied d-bands to higher states of the Fermi level. The peaks found in the range of 357–377 nm are attributed to the band-to-band transition. The blue emission peaks in the range of 430–437 nm as shown in Fig. 7 for the ZnO NWs and ZnO NSCs can be attributed to the O and Zn defects such as interstitial defects or vacancies [19, 26, 29]. In fact, the peak found around the 571 nm for the NSC structure is attributed to the dominance of other intrinsic defects that exists in the ZnO lattice which perturbs its band structure to form a discrete energy level within the band gap. Furthermore, for the ZnO NRs, the green emission peak captured at 508 nm is ascribed to the recombination of a photogenerated hole with the singly ionized charged state of the specific defect [8, 26]. A broad green luminescence band captured at the peak of 550 nm for the ZnO thin film is normally related to the crystallinity of the film [8, 26].

Finally, it can be understood from the spectra shown in Fig. 7 that ZnO NWs and NRs exhibit better emission behaviour under UV excitation as compared to ZnO NSCs and thin film where their visible range emissions are strongly quenched.

### Charged Particle Transport Properties

**Fig. 8:**
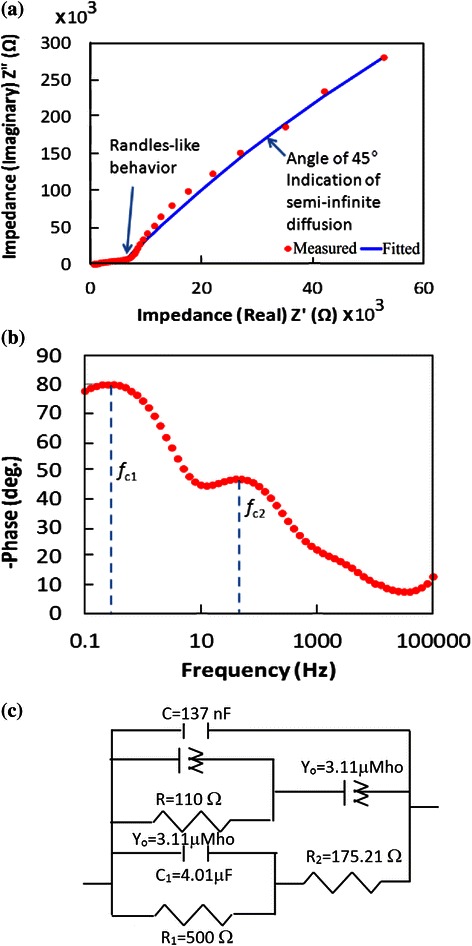
Results of EIS measurements for the ZnO NWs. **a** Nyquist plot, **b** Bode plot and **c** equivalent fitted circuit

The results of EIS measurements for the ZnO NWs on graphene sample are shown in Fig. 8.

By comparing the Nyquist plot (Fig. 8a) to the Bode plot (Fig. 8b), it can be understood that at low frequency range, the electrons transfer via Randles-like behaviour at the graphene/electrolyte interface as represented by a constant phase element of the Randles circuit component *Y*_0_ as shown in the equivalent circuit (Fig. 8c). This conclusion is attributed to the semi-circle that appears in the Nyquist plot just prior to the linear part [30, 31]. The calculated lifetime (*τ*) of an electron through the graphene layer is found to be *τ*_c1_ = 1/2*πf*_c1_ = 3.18 ms [30], where *f*_c_ is the frequency of the charged particle. At middle range of frequency, the electrons tend to transfer at the ZnO/electrolyte interface via semi-infinite length diffusion (represented by capacitance *C*_1_ and resistance *R*_1_ as shown in Fig. 8c [23, 24]). The calculated lifetime of electron through the ZnO structure is found to be 265 ms at *f*_c2_ [30, 31]. The above performance could be more explained by paying attention to the electrochemistry of the measurements, i.e. investigating the reactions in which the charged particles (ions and/or electrons) cross the interface between different phases of matter, such as the interface between electrodes and electrolyte.

**Fig. 9:**
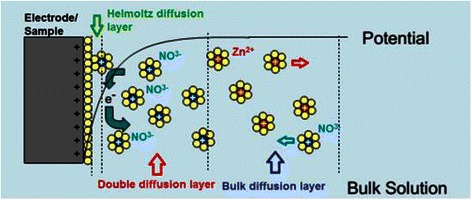
Schematic representation for sample-electrolyte interface based on the stern model

Figure 9 shows a schematic representation for a sample-electrolyte interface based on the stern model [32]. Actually, after the excitation signal is applied, the Zn^2+^ ions are dissociated from the electrolyte and migrate towards the negatively charged anode. On the other hand, the negatively charged particles (NO^3−^ and electrons) migrate towards the positively charged sample. The particle transport at this stage is mainly driven by the electrochemical potential. According to the potential gradient, the particle migration follows three different diffusion patterns as shown in Fig. 9. It can be seen that prior to the attraction, the particles are transported in a bulk diffusion layer (a layer where all kinds of charged particles coexist). The moment the particles are attracted to the electrode (of opposite charge), the particles that started migration should exist in a new diffusion layer (double diffusion layer) where only particles with similar charges coexist. When the particles get close enough to the electrode, they go through the Helmholtz diffusion layer, where the charged particles start to rearrange at the electrode surface and traffic to start diffusion through electrode. The NO^3−^ ion might exist in clusters due to the possible hydration. Thus, the inner Helmholtz plane (IHP) is used to distinguish the rearranged NO^3−^ and electrons from the solvated cations and clusters which exist at the outer Helmholtz plane (OHP) [32–34].

The diffusion through the sample and the electrode starts after the IHP plane is formed. At this stage, the charged particle transport is mainly controlled by electrostatic attraction, transport channel geometry and charge carriers concentration [32–34].

**Fig. 10:**
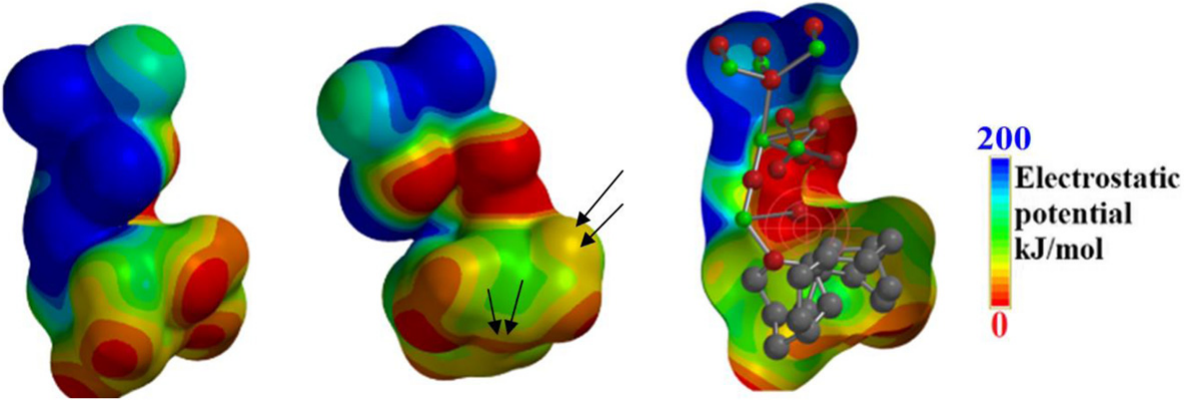
Electrostatic map for the ZnO NWs/graphene hybrid structure. The *blue areas* represent the channels with highest electrostatic potential, whereas the *red areas* represent the forbidden areas for negatively charged particles

Thus, the semi-empirical calculations with MP2 functional is used to calculate the electrostatic potential map through the atomic structure of the ZnO NWs/graphene in order to depict the traffic of the negatively charged particles at the IHP layer and predict the shapes of the transport channels. Figure 10 shows the electrostatic map from two side views. The blue areas represent the channels with the highest electrostatic potential; in other words, it is the area of the highest population of the negatively charged particles. On the other hand, the red areas represent the forbidden areas where the negatively charged particles cannot exist.

As can be seen in Fig. 10, the blue areas dominate the map and are located at the ZnO branches, while the population at the graphene layer is found to be minor. This difference in population can explain why the electron lifetime at the ZnO side of 265 ms can drop to be as low as 3.18 ms. It seems to show that the crowded ZnO channels have less mobile electrons than the graphene layer. Thus, the rate of diffusion towards the graphene is slow; however, after reaching the less crowded graphene surface, the charged particles can move much faster [35]. Furthermore, the red and purple arrows shown in Fig. 10 indicate the long and narrow channels suitable for a finite length diffusion at the ZnO side which explain the Randles-like diffusion shown in the Nyquist plot of Fig. 8a. While, the black arrows show wider and shorter channels suitable for semi-infinite length diffusion at graphene matrix.

**Fig. 11:**
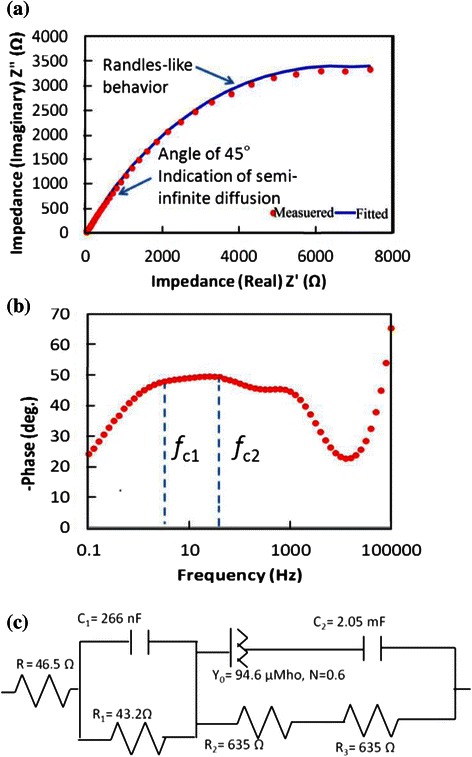
Results of EIS measurements for the ZnO NRs/graphene. **a** Nyquist plot, **b** Bode plot and **c** equivalent fitted circuit

Based on the developed models, we further discuss the results of EIS measurement for ZnO NRs and NSCs and their respective computational analysis. Figure 11 presents the results of EIS measurements for the grown ZnO NRs/graphene structure. Similarly, the results as shown in the Nyquist plot (Fig. 11a) and in the Bode plot (Fig. 11b) indicate that at high frequency range, the electrons transfer via semi-infinite diffusion at the electrode/electrolyte interface (represented by capacitance *C*_1_ and resistance *R*_1_ as shown in equivalent circuit of Fig. 11c. From the middle to low range of frequency, the electrons transfer at the ZnO NRs/electrolyte interface in a Randles-like behaviour (represented by a constant phase element *Y*_0_ as shown in Fig. 11c. The calculated lifetime of electron through the NR is found to be *τ*_c1_ = 1/2*πf*_c1_ = 159 ms while the lifetime of electrons at graphene matrix is found to be 7.95 ms at *f*_c2_ [33, 35].

**Fig. 12:**
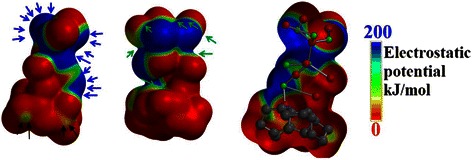
Electrostatic map for the ZnO NRs/graphene hybrid structure. The *blue areas* represent the channels with highest electrostatic potential, whereas the *red areas* represent the forbidden areas for negatively charged particles

Figure 12 shows the electrostatic map for the ZnO NRs/graphene sample from two side views. As can be seen in Fig. 12, the blue areas dominate the map and are located at the ZnO branch.

However, as compared to the potential map of the ZnO NWs/graphene sample, the blue channels are getting smaller. On the other hand, the red iso-surfaces are getting bigger and thus the existence of negatively charged particles at the graphene layer is almost prohibited. This change in channel size clarify the reduction of electron life time at the ZnO side to be 159 ms and the increase of the life time up to 7.95 ms at graphene matrix as compared to the ZnO NWs/graphene. In fact, the reduction of the iso-surfaces of the high electrostatic potential (blue iso-surfaces at electrostatic potential map) indicates the reduction of the number of diffusing particles at the ZnO branch, thus increasing the mobility. The rate of diffusion towards the graphene is increasing, however after reaching the less crowded graphene surface, the charged particles can move much faster. This resulted from the localization of LUMO at graphene matrix which leads to a better charge transfer from graphene side to ZnO end. This results to the reduction of the band gap and the increase of the life time of the transporting charge as discussed in Fig. 6. Furthermore, the green arrows shown in Fig. 12 indicate the long and narrow channels suitable for a semi-infinite length diffusion at the ZnO side which explain the semi-infinite diffusion as shown by the Nyquist plot in Fig. 11a. While, the black arrows show the few separated spots available for finite length diffusion at graphene matrix.

**Fig. 13:**
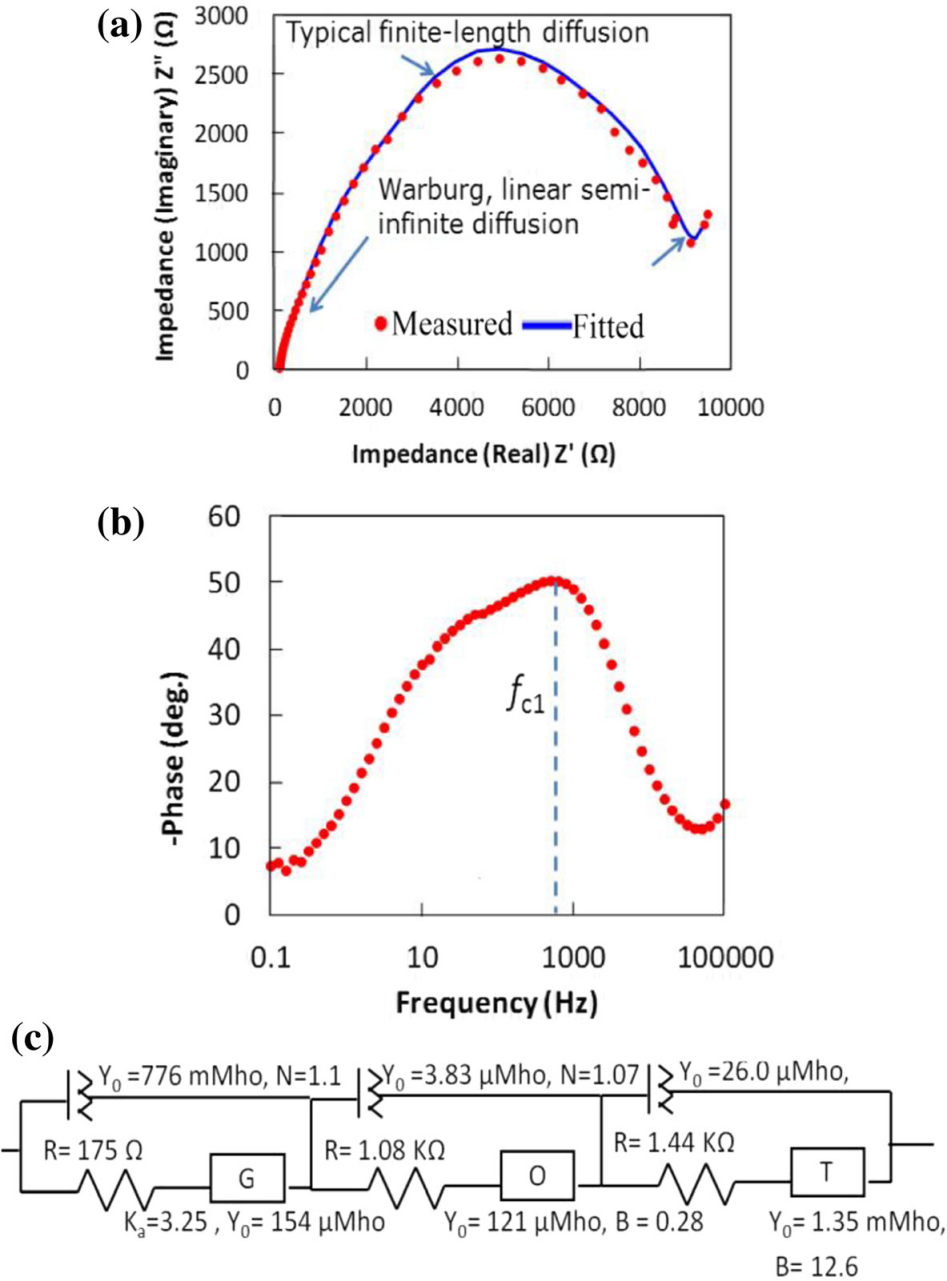
Results of EIS measurements for the ZnO NSCs/graphene. **a** Nyquist plot, **b** Bode plot and **c** equivalent fitted circuit

The EIS measurements results for the grown ZnO NSCs nanostructures on graphene substrate are shown in Fig. 13. By comparing the Nyquist plot (Fig. 13a) to the Bode plot (Fig. 13b), it can be understood that at high frequency range, the electrons transfer via Warburg linear diffusion which is a semi-infinite diffusion that occurs at the electrode/electrolyte interface.

At middle range of frequency, the electrons tend to transfer at the ZnO NSCs/electrolyte interface via finite length diffusion (represented by O element shown in Fig. 13c). The calculated lifetime of electron through the NSCs was found to be *τ*_c1_ = 1/2*πf*_c1_ = 19.89ms. At low range of frequency, electrons transfer through the graphene via hyperbolic tangent diffusion (represented by T-element shown in Fig. 13c.

**Fig. 14:**
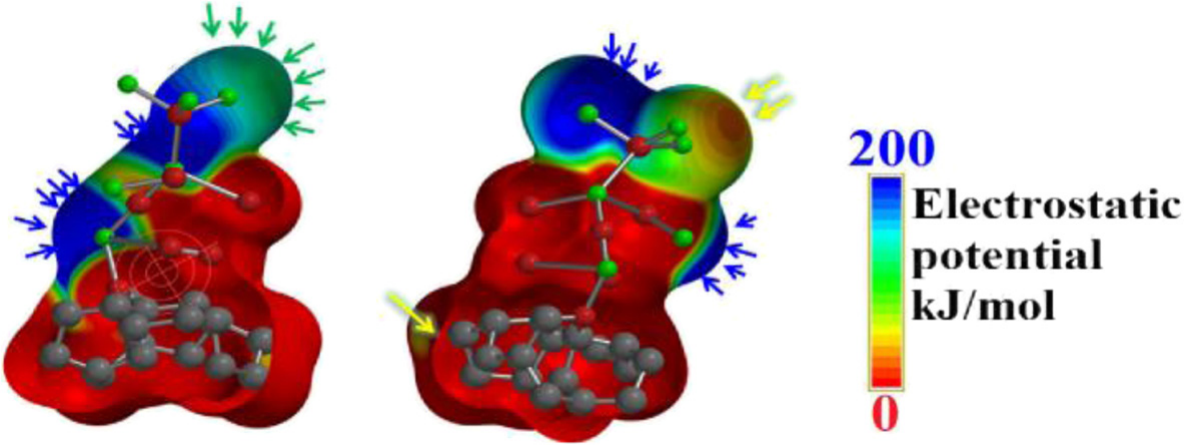
Electrostatic map for the ZnO NSCs/graphene hybrid structure. The *blue areas* represent the channels with highest electrostatic potential, whereas the *red areas* represent the forbidden areas for negatively charged particles

Finally, Fig. 14 shows the electrostatic map for the ZnO NSCs/graphene sample from two side views. As can be seen in Fig. 14, the blue areas at the ZnO branch remarkably shrink and segregated as compared to the potential map of the ZnO NWs/graphene sample. On the other hand, the red iso-surfaces are getting bigger, even extended to the most of the ZnO branch and thus the existence of negatively charged particles at the graphene layer and at the ZnO branch is almost prohibited. These changes in channel size and distribution can verify the reduction of electron lifetime at the ZnO side to 19.89 ms during the diffusion to and at the graphene matrix as compared to the ZnO NWs/graphene sample. In fact, the reduction of the iso-surfaces of the high electrostatic potential (blue iso-surfaces at electrostatic potential map) indicates the reduction of the number of diffusing particles at the ZnO branch; hence, the mobility is dramatically increased.

On the other side, the rate of diffusion towards the graphene is totally prohibited due to the isolation of the charged particles that reach the sites of the ZnO branch by the dominating red areas. This can be attributed to the excited state of S1 as discussed in Fig. 5, where the charges travel from the HOMO orbital that localized at the free end of the ZnO branch to the LUMO orbital that localized at the graphene matrix. Thus, the distance to be travelled by electrons seems to be long and that is the main cause of the large band gap. In other words, the role of the graphene layer is eliminated in some way. Furthermore, the green arrows shown in Fig. 14 indicate the localized isolated channels suitable for a finite length diffusion at the ZnO side which explain the finite length diffusion band shown in the Nyquist plot in Fig. 13a. The blue arrows show few separated spots available for the Warburg infinite length diffusion at the ZnO terminal. Finally, the yellow arrows indicate the possible channel for a hyperbolic diffusion of negatively charged particles.

## Conclusions

In this work, the computational analysis of the measured optical and charge transport properties of the spray pyrolysis-grown ZnO nanostructures was developed. The induced stresses in the lattices of ZnO NSCs that formed during the pyrolysis process seem to cause the narrowing of the gap between the energy levels. On the other hand, ZnO NWs and NRs show homogeneous distribution of the LUMO and HOMO orbitals all over the entire heterostructure. Such distribution contributes to the reduction of the band gap down to 2.8 eV, which has been confirmed to be in a good agreement with the experimental results. It was found that LUMO orbitals are distributed all over the ZnO branch in the NW and NR structures as a result of the excessive O atoms. Furthermore, ZnO NWs and NRs exhibited better emission behaviour under the UV excitation as compared to ZnO NSCs and thin film as their visible range emissions are strongly quenched. The electrostatic potential density map for the ZnO NWs show crowded ZnO channels with less mobile electrons than the graphene layer where the rate of diffusion towards the graphene was found to be small. However, after reaching the less crowded graphene surface, the charged particles move much faster. The electrostatic potential density map for the ZnO NRs indicates the reduction of the number of diffusing particles at the ZnO branch, thus increasing the mobility. The rate of diffusion towards the graphene is increasing; however, after reaching the less crowded graphene surface, the charged particles can move much faster. Finally, for the ZnO NSCs, the number of diffusing particles at the ZnO branch was drastically reduced, thus the mobility is dramatically increased. On the other side, the rate of diffusion towards the graphene is totally prohibited due to the isolation of the charged particles reach cites of the ZnO branch by the dominating red areas. In other words, the role of the graphene layer is eliminated in some way.
